# MicroRNA editing patterns in Huntington’s disease

**DOI:** 10.1038/s41598-022-06970-6

**Published:** 2022-02-24

**Authors:** Shiyong Guo, Jun Yang, Bingbing Jiang, Nan Zhou, Hao Ding, Guangchen Zhou, Shuai Wu, Angbaji Suo, Xingwang Wu, Wenping Xie, Wanran Li, Yulong Liu, Wei Deng, Yun Zheng

**Affiliations:** 1 State Key Laboratory of Primate Biomedical Research, Institute of Primate Translational Medicine, Kunming University of Science and Technology, Kunming, 650500 China; 2 Physical Evidence Spectral Technology Innovation Team, Yunnan Police College, Kunming, 650223 China; 3 Faculty of Information Engineering and Automation, Kunming University of Science and Technology, Kunming, 650500 China; 4 Center of Statistical Research, Southwestern University of Finance and Economics, Chengdu, 611130 China

**Keywords:** Data integration, Genome informatics

## Abstract

Huntington’s disease (HD) is a neurodegenerative disease. MicroRNAs (miRNAs) are small non-coding RNAs that mediate post-transcriptional regulation of target genes. Although miRNAs are extensively edited in human brains, the editome of miRNAs in brains of HD patients is largely unknown. By analyzing the small RNA sequencing profiles of brain tissues of 28 HD patients and 83 normal controls, 1182 miRNA editing sites with significant editing levels were identified. In addition to 27 A-to-I editing sites, we identified 3 conserved C-to-U editing sites in miRNAs of HD patients. 30 SNPs in the miRNAs of HD patients were also identified. Furthermore, 129 miRNA editing events demonstrated significantly different editing levels in prefrontal cortex samples of HD patients (HD-PC) when compared to those of healthy controls. We found that hsa-mir-10b-5p was edited to have an additional cytosine at 5’-end in HD-PC, and the edited hsa-mir-10b repressed GTPBP10 that was often downregulated in HD. The down-regulation of GTPBP10 might contribute to the progression of HD by causing gradual loss of function of mitochondrial. These results provide the first endeavor to characterize the miRNA editing events in HD and their potential functions.

## Introduction

Huntington’s disease (HD) is an incurable hereditary neurodegenerative disorder. Abnormally long and unstable CAG repeats identified in HD patients were intensively studied for many years^1–3^. The pathogenesis of HD has not been fully elucidated, and recently it has been discovered that Huntington (HTT) protein gave few homologies to any known proteins and the mutant HTT protein results in a GOF (gain of function) mechanism and neuronal death^4^. In particular, gene expressions in neuronal and non-neuronal cells have severely changed in HD patients. A number of transcriptional regulators such as REST (Repressor element-1 transcription factor)^5^, TBP (TATA-Box Binding Protein)^6^, CBP (CREB-binding Protein)^7,8^, RE1 (Repressor element-1)^9^ and P53^7,10^ are known to interact not only with HTT, but also with small non-coding RNAs such as miRNA^11^. Dysregulation of miRNA may also affect the CAG length and the progression of HD^12,13^.

MicroRNA(miRNA) is transcribed by RNA polymerase II (Pol II), then cleaved by DGCR8/DROSHA (DiGeorge syndrome critical region gene 8 / Drosha Ribonuclease III) to produce a hairpin structured precursor with around 70 nucleotide (nt) in length, which is then exported to cytoplasm by exportin 5^14^. In cytoplasm, Dicer makes a second cleave to remove the loop end of the precursor and produce a miRNA:miRNA* duplex^14^. miRNA is then loaded into RNA induced silencing complex (RISC) with an argonaute protein and guides the RISC to its target mRNA (messenger RNA) through its complementary site on target mRNA^14,15^. miRNA can lead to silencing of its target mRNA either via inhibition of translation or degradation of the mRNA^13,15^. Furthermore, miRNAs were reported as important regulators in many biological processes including cell proliferation, differentiation, development, metabolism and so on^16^.

Recent studies had shown the essential roles of miRNAs in regulation in diverse neurological and neurodegenerative disorders, such as Parkinson’s disease, Huntington’s disease^17–19^. The altered miRNA expression and the potential role on the altered mRNA expression have been reported in cellular model of mice and human HD samples with microarray technologies and next generation sequencing^20^. Five miRNAs (miR-10b-5p, miR-196a-5p, miR-196b-5p, miR-615-3p and miR-1247-5p) were upregulated in prefrontal cortex of HD patients^21^. Recent analysis had shown that post-transcriptional regulation of gene expression by miRNAs forms a network in HD. For example, miR-22 is associated with protective mechanisms against Huntington’s disease^22^. miR-22 possibly interacts with three genes that implicated in the pathogenesis of HD, including histone deacetylase 4 (HDAC4), REST corepressor 1 (RCOR1) and regulator of G protein signaling 2 (RGS2)^9,23–26^.

MiRNAs are edited in several ways, including 3’ non-templated addition^27–30^, Adenosine-to-Inosine (A-to-I) editing performed by adenosine deaminase (ADAR)^31–36^ and C-to-U (Cytidine-to-uridine) editing performed by apolipoprotein B mRNA editing polypeptide-like (APOBEC) enzymes^33–36^. Editing of miRNAs played a key role during development, and miRNAs generally showed low levels of editing before birth that increased until adulthood^37,38^. Many studies have reported that changes in miRNA editing could both play a role in tumor pathogenesis and progression, and be used as early and specific biomarkers of selected forms of cancer, as reviewed in literature^39–41^. For examples, Choudhury et al. demonstrated that the unedited miR-376a* promoted glioma cell migration and invasion in 2012, while the edited miR-376a* suppressed these features^42^. The original miR-455-5p is highly expressed in malignant melanoma and silences cytoplasmic polyadenylation element binging protein 1 (CPEB1), a tumor suppressor^43^. The editing of miR-455-5p was mediated by ADAR1^43^, which is downregulated in metastatic melanoma compared to benign or intermediate stages of the diseases^44^. The reduced editing level of miR-455-5p contributes to the upregulation of the original miR-455-5p, which enhances melanoma growth and metastasis^43^. In hepatocellular carcinoma, miRNA editing events cause a decrease in RNA transcripts complementary to pri-miR-214, which leads to a decrease in pri-miR-214 and miR-214 and leads to an increase in protein levels of its new target gene Rab15^45^. Nigita et al. detected disordered miRNA editing events between non-small cell lung cancer tumors and normal tissues, and found that miR-411-5p editing occurring at position 5 was significantly disordered in tissues and exosomes of patients with non-small cell lung cancer^46^. Furthermore, it had been reported that A-to-I editing in miRNAs had higher editing levels in brain than in other organs^38,47,48^.

Single nucleotide polymorphism (SNP) is a common type of single base variation in DNA sequences in the human genome, which is an important variation of inter-individual diversity and an important cause of phenotypes, traits and diseases^49–52^. SNPs in miRNA genes can affect the function of them by modulating the transcription of the primary transcripts, processing of pri-miRNAs and pre-miRNAs, maturation, or miRNA:target interactions^52–55^. Duan et al. reported that an SNP in miR-125a significantly blocked the processing of pri-miRNA to pre-miRNA, in addition to reducing miRNA-mediated translational suppression^55^. Many studies have reported that SNPs in some miRNA genes are associated with human diseases, such as breast cancer^56–58^, chronic lymphocytic leukemia^59^, papillary thyroid carcinoma^60^ and progressive hearing loss^61^.

However, the miRNA editing patterns in Huntington’s disease are largely unknown. In this research, to comprehensively characterize miRNA editing sites in brain tissues in HD, we systematically analyzed 28 and 83 small RNA sequencing profiles of brain tissues of HD patients and normal people, respectively. We have identified 1182 miRNA mutation and/or editing (M/E) sites that have significantly editing levels in HD patients. Furthermore, 129 M/E sites with significantly different editing levels in the prefrontal cortex regions of HD patients were identified. One 5’-editing site on hsa-mir-10b-5p introduced an additional cytosine to its 5’-end, which severely changed the targets of edited hsa-mir-10b-5p. Among 308 new targets of edited hsa-mir-10b-5p, GTPBP10 (GTP binding protein 10) was downregulated in HD, which potentially deteriorated HD. These results represented the first view of miRNA editome in HD and increased our understanding of miRNA editing in HD.

## Results

### Summary of the miRNA M/E sites identified in HD

To comprehensively identify miRNA M/E sites in HD, we collected 111 sRNA-seq (Small RNA sequencing) profiles in postmortem HD patients and healthy controls including 28 prefrontal cortex (Brodmann Area 9, BA9) regions of postmortem HD patients (HD-PC) and 83 samples of healthy people, with 38 prefrontal cortex (BA9) (PC), 14 amygdala (Am), 6 frontal cortex (FC), 6 corpus callosum (CC), 3 astrocytes (As), 1 temporal neocortex gray matte (NG) and 15 unknown region (Unknown) (see Table S1).

**Figure 1:**
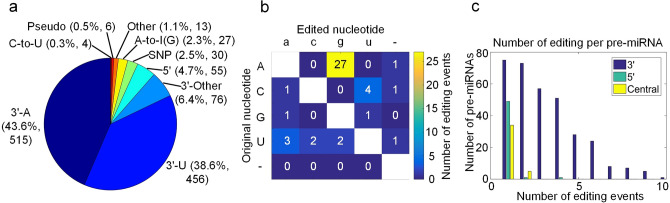
A summary of miRNA mutation and editing sites. (**a**) The categories of significant M/E sites in miRNAs. (**b**) The numbers of different types of editing sites in the central regions of miRNAs. (**c**) The distributions of the numbers of pre-miRNAs with editing events happened at 3’-, 5’-ends and central regions of mature miRNAs.

We utilized the MiRME (an algorithm for identifying miRNA Mutation and Editing sites) pipeline to analyze the 111 sRNA-seq profiles selected with default settings. Totally, we found 1182 M/E sites with significant editing levels in these samples (Fig. 1a and Table S3). The largest category of these M/E sites are 3’-A, accounting for 43.6% with 515 sites, then followed with 3’-U, which accounts for 38.6% with 456 sites, and 3’-Other (6.4% with 76 sites) (Fig. 1a). A-to-I and C-to-U editing account for 2.3% with 27 sites and 0.3% with 4 sites, respectively (Fig 1a). And 30 SNPs (Single Nucleotide Polymorphism) were found after we compared the identified M/E sites to reported SNPs in miRNAs (see Materials and methods). Other types of editing accounts for 1.1% with 13 sites (see Fig. 1a).

The M/E sites of A-to-I, C-to-U and Other types not at the 3’ and 5’ end of miRNAs were further investigated based on the bases variation types. 27 A-to-I editing events and 4 C-to-U editing events were detected, and 13 other editing events were also classified. Moreover, 3 nucleotides of insertion or deletion were found in these 111 samples (see Fig. 1b).

Furthermore, we have counted numbers of the editing events happened on the 5’, 3’ end and central regions of the pre-miRNAs (precursor miRNAs). Results showed that editing events could happen on the different positions of the pre-miRNAs whose nucleotides could be edited by substitution or insertion during the maturation. Interestingly, along with edited numbers of pre-miRNA increasing, the frequency of editing events happened at 3’ ends was higher compared with it happened at 5’ ends and central regions. Although a few editing events were observed on the 5’ ends of pre-miRNAs, 3 editing events may happen at several positions, and only one or two editing events were detected in the central regions of mature miRNAs (see Fig. 1c).

### A-to-I editing sites

**Figure 2:**
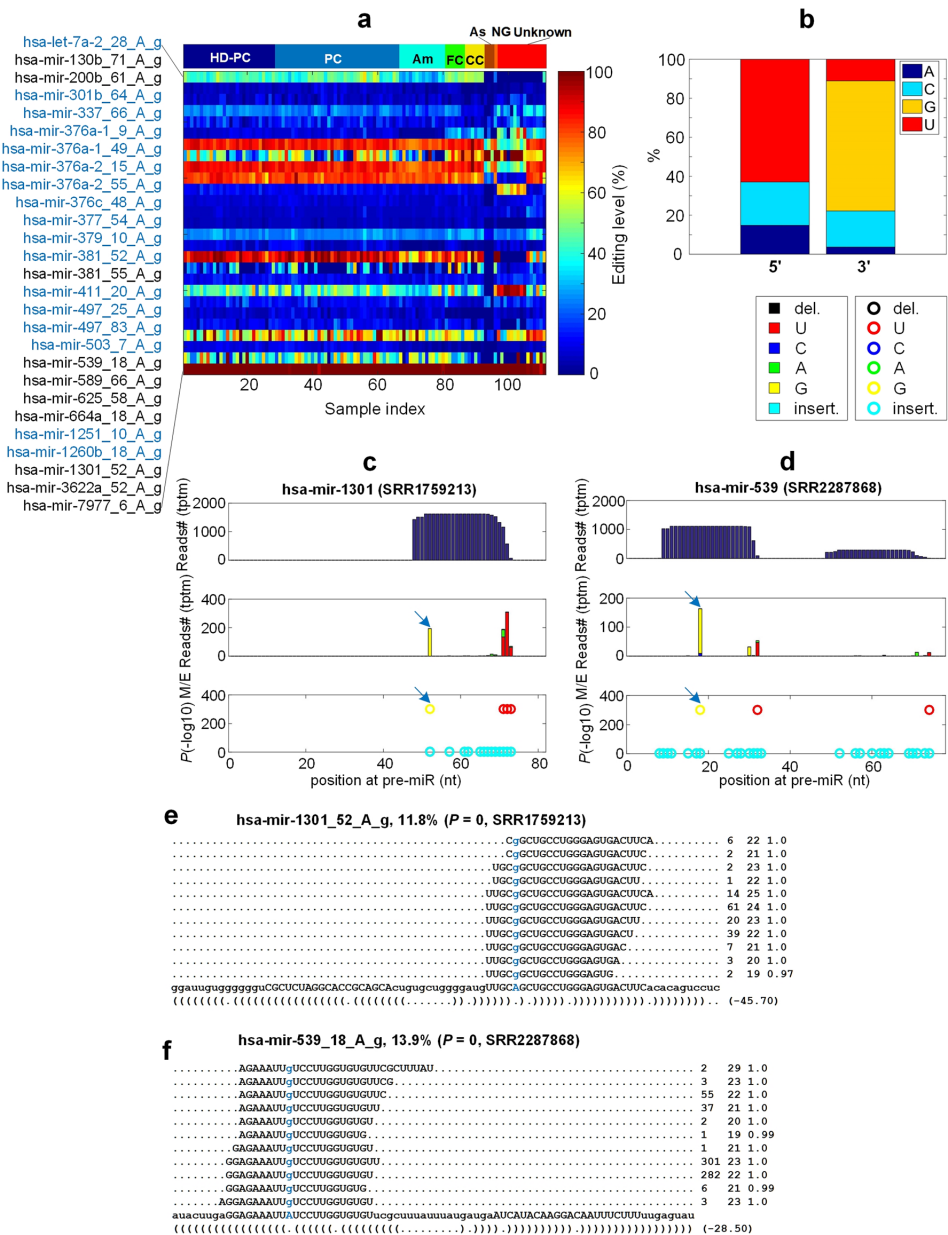
The detailed information of A-to-I editing sites in miRNAs of HD samples. (**a**) The editing levels of 27 A-to-I editing sites in 111 selected sRNA profiles. The tissues from left to right are prefrontal cortex (BA9) regions of HD patients (HD-PC), prefrontal cortex (BA9) regions of healthy controls (PC), amygdala regions of healthy controls (Am), frontal cortex regions of healthy control (FC), corpus callosum regions of healthy controls (CC), astrocytes of healthy controls (As), temporal neocortex gray matter region from healthy control (NG) and unknown regions from healthy controls (Unknown). The sites with blue names are conserved in mammals. (**b**) The base distribution of A-to-I editing sites at 5’ and 3’ end of the mature miRNAs. (**c**) The MiRME map of hsa-mir-1301 in one of the PC samples selected (SRR1759213). The three panels from top to bottom displayed the total number of reads (in Tags Per Ten Million sequencing reads, tptm), the number of M/E reads with variations (in tptm), and *P*-values of the M/E events shown in the central panel, respectively. (**d**) The MiMRE map of hsa-mir-539 in one of the FC samples selected (SRR2287868). (**e**) The details of hsa-mir-1301_52_A_g in SSR1759213. (**f**) The details of hsa-mir-539_18_A_g in SRR2287868. In Part (e)-(f), the edited nucleotides are shown in blue. And the first number on the right indicates the number of raw sequencing reads, the second number indicates the length of the reads, and the third number indicates the weight of the reads on this locus as calculated by the cross-mapping correction algorithm^84^.

We totally found 27 A-to-I editing sites with significant editing levels in the 111 samples used in this study (Fig 2a). Among them, 17 sites (marked in blue) were conserved among human, monkey and/or mouse when compared with reported A-to-I editing sites in monkey and/or mouse^31,36,47,62^ (Fig. 2a and Table S4). According to the TPTM (Tags Per Ten Million sequencing reads) and M/E editing percent of these 27 A-to-I editing sites, 6 highly edited sites were found, which were hsa-mir-411_20_A_g, hsa-mir-381_52_A_g, hsa-mir-381_55_A_g, hsa-mir-376a-1_9_A_g, hsa-mir-376c_48_A_g, hsa-mir-379_10_A_g (see Fig. 2a and Table S4).

After statistical analysis of nucleotides besides these A-to-I editing sites, we found that the 5’ and 3’ end of these A-to-I editing sites preferred to be U and G (Fig. 2b), respectively, which was also noticed in previous studies^36,47,48,62^. As examples, MiRME maps of hsa-mir-1301 and hsa-mir-539 in one of the prefrontal cortex and frontal cortex samples (SRR1759213 and SRR2287868, respectively) were presented in Figs. 2c and 2d. As shown in Figs. 2e and 2f, these two A-to-I editing sites were supported by a lot of sequencing reads in these samples.

### C-to-U editing sites

**Figure 3:**
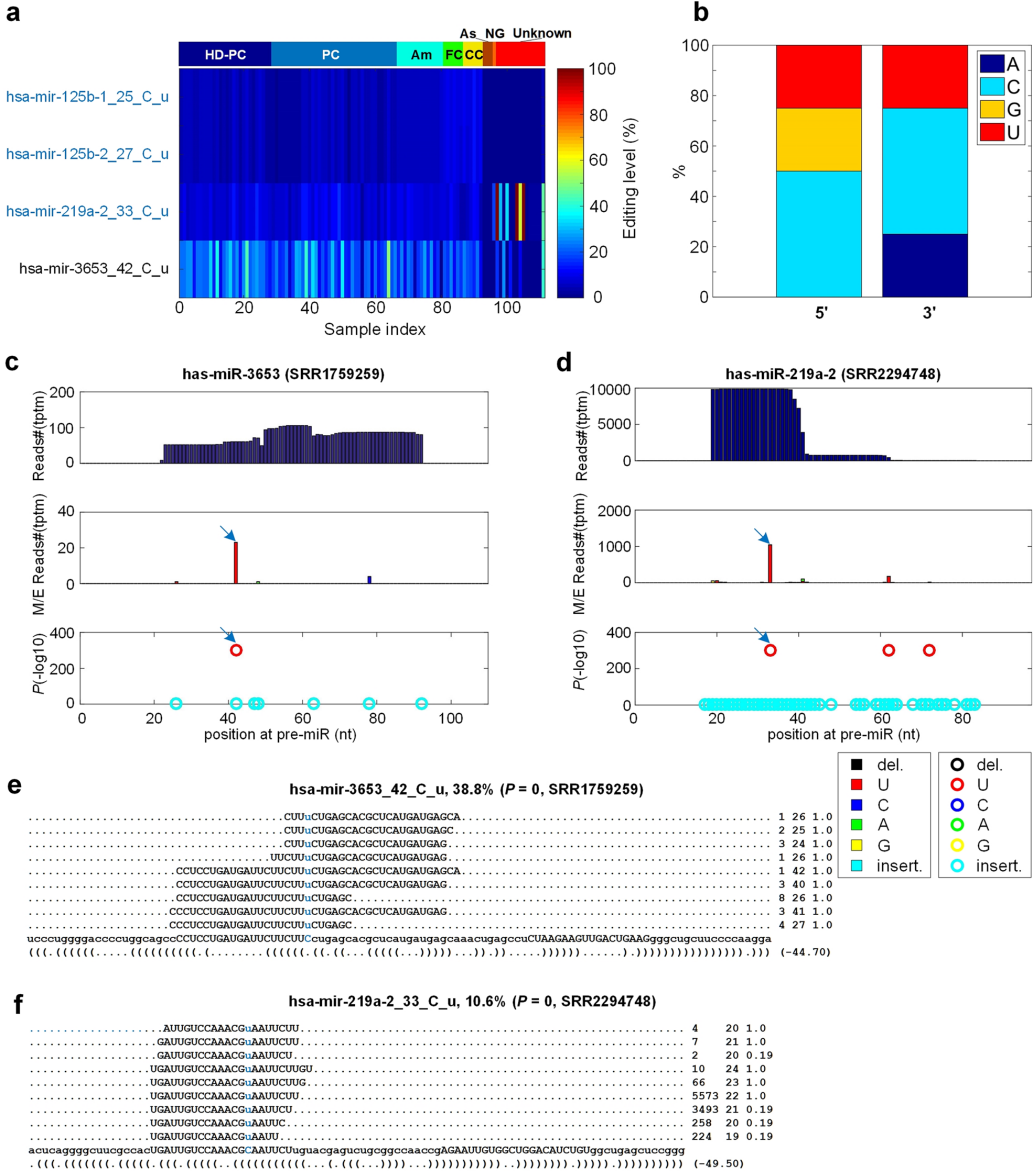
The detailed information of C-to-U editing sites in miRNA of HD samples. Legends are the same as those in Fig. 2. (**a**) The editing levels of 4 C-to-U sites in the 111 selected sRNA-seq profiles. (**b**) The base distribution of C-to-U sites at 5’ and 3’ end of mature miRNA. (**c**) MiRME map of hsa-mir-3653 in one of the HD-PC samples selected (SSR1759259). (**d**) MiRME map of hsa-mir-219a-2 in one of the Am samples selected (SSR2294748). (**e**) The details of hsa-mir-3653_42_C_u in SRR1759259. (**f**) The details of hsa-mir-219a-2_33_C_u in SRR2294748. In Part (e)-(f), the edited nucleotides are shown in blue. And the first number on the right indicates the number of raw sequencing reads, the second number indicates the length of the reads, and the third number indicates the weight of the reads on this locus as calculated by the cross-mapping correction algorithm^84^.

We carefully investigated the 4 C-to-U editing sites through the MiRME pipeline^36^ (as shown in Fig. 3a and Table S5). Three of them (marked in blue) were conserved in primates as previous reported^62^, suggesting that C-to-U editing is a conserved mechanisms with unknown functions. Moreover, both the 5’ and 3’ side of these C-to-U editing sites preferred to be C (Fig. 3b), which was accordant with CCC motif of APOBEC3G (apolipoprotein B mRNA editing enzyme catalytic subunit 3G)^63,64^. And two examples of the C-to-U editing sites were shown in Fig. 3c and d, the detailed editing sites and editing levels of these two miRNAs were also shown in Fig. 3e and f. Editing events on these two sites were supported by several hundreds of reads suggesting good reliability.

### Insertion/Deletion events in miRNAs

There are some insertion and deletion editing events in some miRNAs and their precursors in human brain, testis, heart and kidney samples of during their biogenesis process^36,65,66^. In this study, 1182 significant M/E sites were found in 111 human brain samples. There are 44 important editing events that occurred in the central region of miRNA were detected. Among these M/E sites, three deletion events, one A-deletion, one C-deletion, and one U-deletion, respectively, were identified. However, we did not found insertion sites, unlike a few insertion sites identified in our previous study^36^.

### Identified SNPs in miRNAs

**Figure 4:**
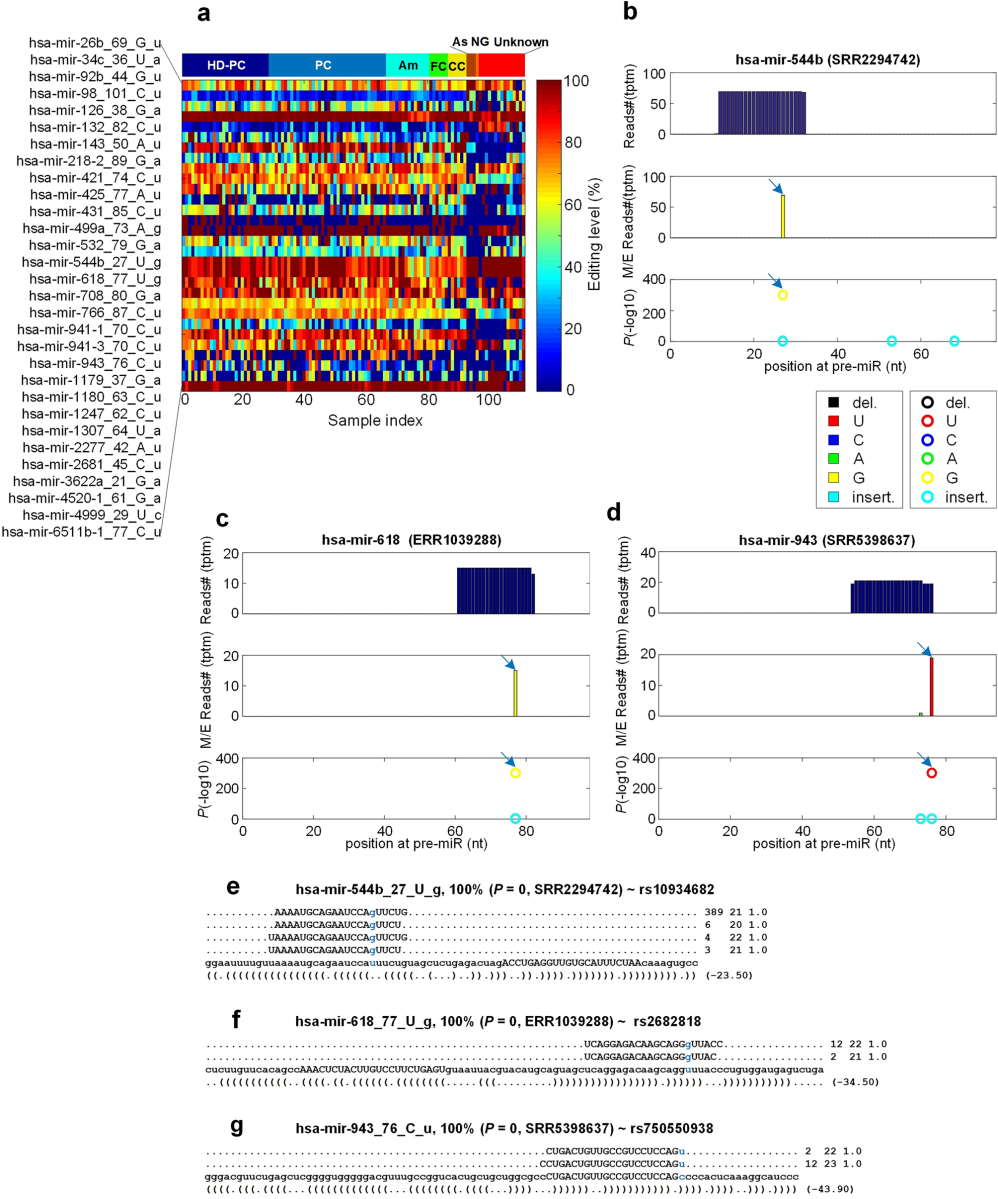
The details of 30 SNPs identified in miRNAs of HD patients. Legends are the same as those in Fig. 2. (**a**) The editing/mutation levels of the 30 SNP sites in the 111 selected sRNA-seq profiles. (**b**) The MiRME map of hsa-mir-544b in one of the CC samples selected (SRR2294742). (**c**) The MiRME map of hsa-mir-618 in one of the Unknown samples selected (ERR1039288). (**d**) The MiRME map of hsa-mir-943 in one of the Am samples selected (SRR5398637). (**e-g**) The details of hsa-mir-544ba_27_U_g in SSR2294742, hsa-mir-618_77_U_g in ERR1039288 and hsa-mir-943_76_C_U in SRR5398637, respectively. In Part (e)–(g), the edited nucleotides are shown in blue. And the first number on the right indicates the number of raw sequencing reads, the second number indicates the length of the reads, and the third number indicates the weight of the reads on this locus as calculated by the cross-mapping correction algorithm^84^.

By comparing the M/E sites to SNPs in dbSNP (Single Nucleotide Polymorphism Database, v151) and examining their editing levels, 30 sites were regarded as SNPs (as shown in Fig. 4a and Table S6) according to the criteria reported previously^62^. Three examples of SNPs in miRNAs were shown in Fig. 4b–d. The editing levels of these three sites are 100% in these three samples (Fig. 4e–g).

From the 30 SNP types of sites we identified, we found that two SNPs, hsa-mir-499a_73_A_g and hsa-mir-3622a_21_G_a (i.e., rs3746444 and rs66683138, respectively), occurred in the seed regions of mature miRNAs. Therefore, we used the MiCPAR algorithm^67^ to predict the targets of these two miRNAs orignal and mutated sequences. The results showed that the mutated miRNA hsa-mir-499a_73g gained 321 new unique targets compared with the original hsa-499a-3p, and the mutated miNRA hsa-mir-3622a_21a obtained 1298 new unique targets compared to the original hsa-mir-3622a-5p (Fig. S2, Table S7). These results showed that these SNPs in miRNAs severely changed the targets of miRNAs.

### Relevant miRNA M/E sites in HD

**Figure 5:**
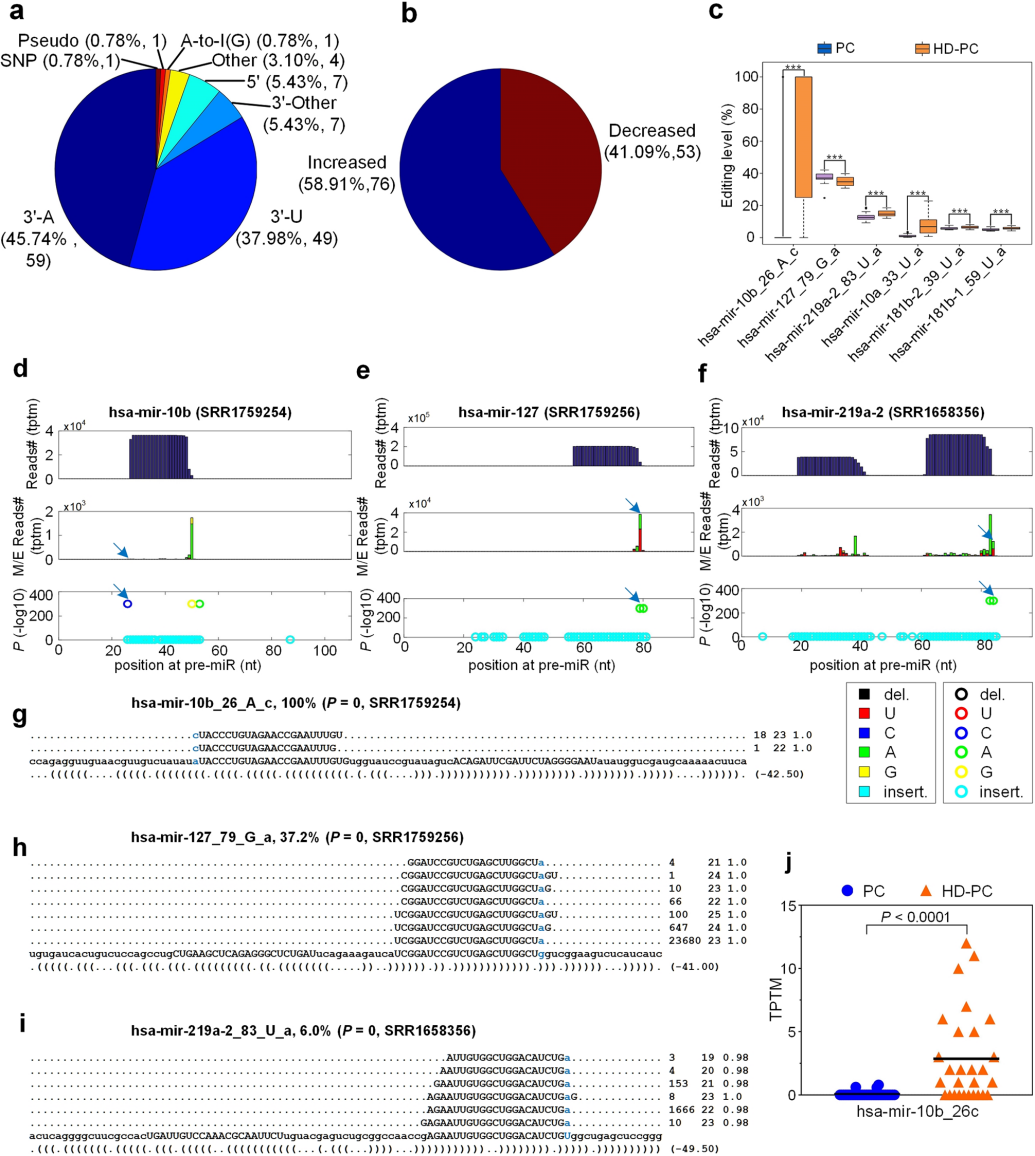
The 129 miRNA M/E sites that have significantly different editing levels in HD-PC samples when compared to healthy controls. (**a**) The categories of the 129 M/E sites. (**b**) The change of editing levels of 129 M/E sites in HD-PC samples compared to PC samples. (**c**) Then editing levels of six miRNA M/E sites that have significantly different editing levels in HD-PC samples when compared to PC samples. “***” indicate *P*-values smaller than 0.05, Mann-Whitney *U*-tests and corrected with the Benjamini and Hochberg method^85^. (**d**) The MiRME map of hsa-mir-10b in one of the HD-PC samples (SRR1759254). (**e**) The MiRME map of hsa-mir-127 in one of the HD-PC samples (SRR1759256). (**f**) The MiRME map of hsa-mir-219a-2 in one of the Unknown samples (SRR1658356). (**g-i**) The details of hsa-mir-10b_26_A_c in SSR1759254, hsa-mir-127_79_G_a in SSR1759256 and hsa-mir-219a-2_83_U_a in SSR1658356, respectively. The edited nucleotide are shown in blue. And the first number on the right indicates the number of raw sequencing reads, the second number indicates the length of the reads, and the third number indicates the weight of the reads on this locus as calculated by the cross-mapping correction algorithm^84^. (**j**) Comparisons of normalized expression levels (in TPTM) of hsa-mir-10b_26c, i.e., hsa-mir-10b-5p with additional cytosine at 5’ end, in the HD-PC and PC samples.

We obtained 129 miRNA M/E sites with significantly different editing levels in Prefrontal Cortex samples of HD patients (HD-PC, n = 28) and control samples (PC, n = 38) with *P*-values smaller than 0.05, Mann-Whitney *U*-tests (Table S8). As shown in Figure 5a and 5b, all the 129 editing sites were divided into 8 categories, among which 3’ end editing events accounts for 89.15% with 115 sites, including 3’-A, 3’-U and 3’-Other editing, A-to-I(G) editing events accounts for 0.78% with only one site, Other Type accounts for 3.10% with 4 sites, but no C-to-U editing events were identified (Fig. 5a). Moreover, most of the 129 sites have increased editing levels in HD-PC samples(58.91% with 76 sites), and 41.09% with 53 sites have decreased editing levels in HD-PC samples (Fig. 5b and Table S8), suggesting that the clinical condition of HD may tend to promote the up-regulation of some related miRNA editing levels in human brains. Furthermore, using the criteria of FDR (False Discovery Rate) value less than 0.05, seven edit events were discovered (Table S9). Except for the pseudo site of hsa-mir-135b_26_C_u , the remaining six sites included four 3’-A (hsa-mir-127_79_G_a, hsa-mir-181b-1_59_U_a, hsa-mir-181b-2_39_U_a, hsa-mir-219a-2_83_U_a), one Other (hsa-mir-10a_33_U_a) and one 5’-editing (hsa-mir-10b_26_A_c). Of these 6 editing sites, only the editing level of hsa-mir-127_79_G_a was significantly reduced in the HD-PC samples (Fig. 5c), and the editing level of the remaining 5 sites (hsa-mir-10a_33_U_a, hsa-mir-10b_26_A_c, hsa-mir-181b-1_59_U_a, hsa-mir-181b-2_39_U_a, hsa-mir-219a-2_83_U_a) was significantly increased in the HD-PC samples (Fig. 5c). The details of three of the 6 editing sites are shown in Fig. 5d–i. Among the editing sites with different levels in HD, hsa-mir-10b_26_A_c, a 5’ editing site (Fig. 5d and g), has much higher editing level in HD-PC samples compared to PC samples (Fig. 5c). And the edited form of hsa-mir-10b-5p (hsa-mir-10b_26c) also had significantly higher expression levels in HD-PC samples compared to PC samples (Fig. 5j). As noticed previously^36^, the 5’ editing site has a preference of C, suggesting that an unknown mechanism is activated in HD and contributes to the adding of additional C at 5’ end of hsa-mir-10b-5p.

### Functional analysis of hsa-mir-10b_26_A_c

**Figure 6:**
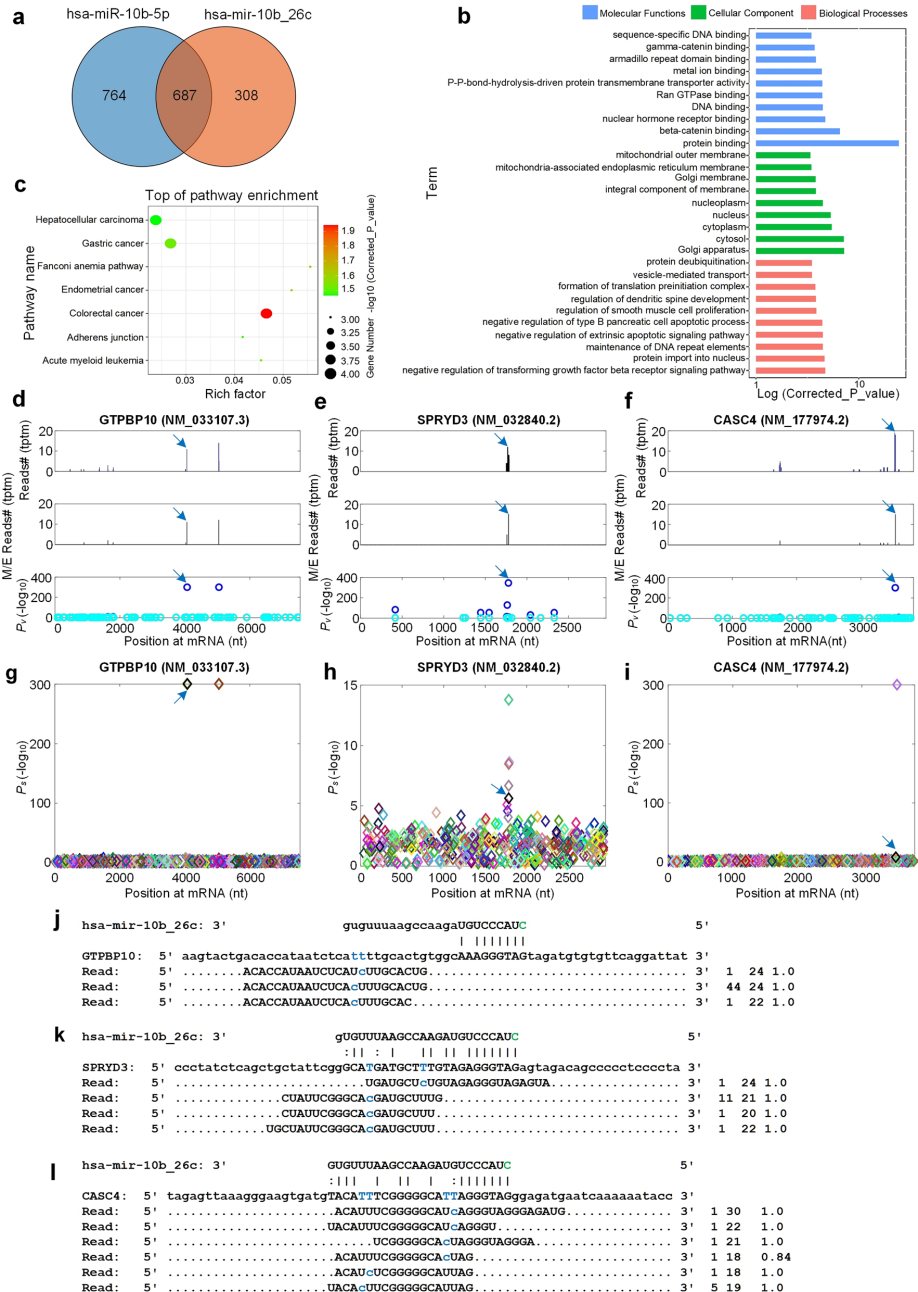
The targets, enriched GO terms, and enriched KEGG pathways of hsa-mir-10b_26c. (**a**) The targets of the original and edited hsa-mir-10b. (**b**) The 10 most significant GO terms of the new targets of hsa-mir-10b_26c. (**c**) The enriched KEGG pathways of the new targets of hsa-mir-10b_26_c. The 10 most significant items with the smallest *P*-values in each of three major GO categories, i.e., Molecular Function, Cellular Pathway and Biological Process, were presented. (**d-f**) The distributions of PAR-CLIP reads on GTPBP10 (NM_033107.3), SPRYD3 (NM_032840.2) and CASC4 (NM_177974.2), respectively. The peaks pointed by blue arrows were the complementary sites of hsa-mir-10b_26c. (**g-i**) The identified miRNA sites and their $P_s$ values on GTPBP10 (NM_033107.3), SPRYD3 (NM_032840.2) and CASC4 (NM_177974.2), respectively. The sites pointed by blue arrows were the complementary sites of hsa-mir-10b_26c. (**j-l**) The details of complementary sites of hsa-mir-10b_26c and PAR-CLIP reads on GTPBP10 (NM_033107.3), SPRYD3 (NM_032840.2) and CASC4 (NM_177974.2), respectively. In Part (j) to (l), the T-to-C nucleotides are shown in blue on mRNAs and PAR-CLIP sequencing reads. The green Cytosine on 5’ end of hsa-mir-10b_26c is the additional cytosine introduced by the RNA editing event, hsa-mir-10b_26_A_c. And the first number on the right indicates the number of raw sequencing reads, the second number indicates the length of the reads, and the third number indicates the weight of the reads on this locus as calculated by the cross-mapping correction algorithm^84^.

We identified the targets of the original and 5’ edited hsa-mir-10b-5p (hsa-mir-10b_26c) with the MiCPAR algorithm^67^ (as listed in Table S10 and S11, respectively). As shown in Fig. 6a, the original and edited hsa-mir-10b-5p shared 687 common targets and 308 new targets of hsa-mir-10b_26c were found (Table S12). Among the 308 newly gained targets, duplicate genes and non-coding genes were removed and 7 targets with at least 10 PAR-CLIP reads were kept for further analysis. They are TMED2 (NM_006815.3, transmembrane p24 trafficking protein 2), GTPBP10 (NM_033107.3, GTP binding protein 10), GOLT1B (NM_016072.4, golgi transport 1B), SPRYD3 (NM_032840.2, SPRY domain containing 3), ZNF503 (NM_032772.5, zinc finger protein 503), TMEM41B (NM_015012.3, transmembrane protein 41B) and CASC4 (NM_177974.2, Cancer susceptibility candidate 4) (Table S12). The distributions of PAR-CLIP reads on GTPBP10, SPRYD3 and CASC4 in Figs. 6d–f indicated that there were clear accumulation of PAR-CLIP reads at the complementary sites of hsa-mir-10b_26c. The identified miRNA complementary sites and their *P-s* values on GTPBP10, SPRYD3 and CASC4 were shown in Fig. 6g–i, respectively. The details of complementary sites of hsa-mir-10b_26c and PAR-CLIP reads on GTPBP10, SPRYD3 and CASC4 were also shown in Fig. 6j–l.

To attempt to understand the potential functional impact of higher expression level of hsa-mir-10b_26c in HD, gene ontology (GO) enrichment analysis was performed for the 308 new targets of hsa-mir-10b_26c with KOBAS (KEGG Orthology Based Annotation System, version 3)^68^ (Table S13). Top GO terms included “protein binding”, “beta-catenin binding”, “Golgi apparatus”, “cytosol”, “negative regulation of transforming growth factor beta receptor signaling pathway” and “regulation of dendritic spine development”, as shown in Fig. 6b. Also, the enriched KEGG (Kyoto Encyclopedia of Genes and Genomes) pathway analysis was performed and the 7 most significant pathways were presented, where “Colorectal cancer” was the most enriched pathway, as shown in Fig. 6c and Table S14.

These analyses suggested that the new targets of hsa-mir-10b_26c might be related to cancer related functions, mitochondrial function and neural development.

## Discussion

After analyzing 28 and 83 sRNA-seq profiles of HD patients and healthy controls, 1182 M/E sites with significant editing levels were identified, including 27 A-to-I, 4 C-to-U, 515 3’-A, 456 3’-U, 76 3’-Other, 13 Other, 55 5’-editing sites, 30 SNPs and 6 pseudo sites. This is consistent with the conclusion reported in our previous study^36,62^ that most editing events of miRNAs usually occur at the 3’ end of mature miRNAs.

Our analysis revealed that 129 M/E sites showed significantly different editing levels in prefrontal cortex samples of HD patients when compared with prefrontal cortex samples of the healthy controls. Most of these M/E sites were also 3’-editing sites. Only one classical C-to-U editing site was identified, and no A-to-I editing site was found. 76 and 53 of these 129 M/E sites showed increased and decreased editing levels in HD-PC samples (Fig. 5), suggesting that the clinical condition of HD tends to promote the editing levels of some miRNA editing sites in the brains of HD patients.

miR-10b-5p had significantly higher expression level in HD^11,21^. The expression of miR-10b-5p was related to huntingtin CAG repeat size, onset age, age at death^11,21^ and striatal involvement^11^. But the role of miR-10b-5p in HD is still unclear. Overexpression of miR-10b-5p in PC12 HTT-Q73 cells increased survival, suggesting the activation of miR-10b-5p in HD may have a neuroprotective role. In addition, miR-10b-5p putatively repressed HTT^11^. On the other hand, miR-10b-5p also repressed brain-derived neurotrophic factor (BDNF)^69^, a growth factor required for the survival and differentiation of striatal neurons, indicating a harmful role of miR-10b-5p to neuronal cells. However, miR-10-5p, and several deregulated miRNAs in HD, such as miR-196a-5p, miR-196b-5p and miR-615-3p, were implicated in nervous system development^11,21^.

In this research, we found that 5’ edited hsa-mir-10b-5p had higher expression level in HD-PC samples (Fig. 5j). Since hsa-mir-10b_26_A_c was enhanced in HD and located immediate before the first nucleotide of hsa-mir-10b-5p, we compared the targets of the original hsa-mir-10b-5p and edited hsa-mir-10b-5p (hsa-mir-10b_26c). We found that hsa-mir-10b_26c may target as many as 308 additional targets, including GTPBP10. GTPBP10 is required for mitochondrial gene expression^70^. Deletion of GTPBP10 will decrease the level of mtLSU (mitochondrial large subunit) and mtSSU (mitochondrial small subunit), while the virtual deletion of 55S monoribosomes will completely prevent the synthesis of mitochondrial proteins^71^. Human OBG (GTPase Obg) protein GTPBP10 binds specifically to large mitochondrial subunits during late maturation, and GTPBP10 is in a complex with other mtLSU biogenesis factors including mitochondrial RNA granule components, the 16S rRNA module and late mtLSU assembly factors such as MALSU1 (mitochondrial assembly of ribosomal large subunit 1), SMCR7L (Smith-Magenis syndrome chromosome region, candidate 7-like), MTERF4 (mitochondrial transcription termination factor 4) and NSUN4 (NOP2/Sun RNA methyltransferase 4)^70^. A mechanistic link between cellular energetic defects and the pathogenesis of Huntington’s disease (HD) has long been hypothesized based on the observations of progressive weight loss in patients and metabolic defects in brain and muscle^72^. Identification of respiratory chain deficits in HD postmortem brain led to the use of mitochondrial complex II inhibitors to generate acute toxicity models that replicate aspects of HD striatal pathology in vivo^72^. The mitochondrial dysfunction may contribute to the localized hypometabolism and progressive atrophy of the HD patients^73^. In this research, we found that an non-templated C was added to 5’-end of hsa-mir-10b-5p in HD-PC samples, which introduce as many as 308 new targets. One of these targets was GTPBP10 that had lower expression in HD-PC samples (Fig. S1), suggesting that hsa-mir-10b_26_A_c may play a role in HD^74^.

In summary, we produced the first view of miRNA editing in HD and increased our understanding of miRNA editing events in HD, and may provided a new perspective for the treatment of HD. In the future, it is interesting to explore whether the miRNA M/E events in HD brain tissues identified in this study could also be detected in human blood. Because it is convenient to detect miRNA M/E events in blood, and edited miRNAs, such as edited hsa-mir-10b_26c in Fig. 5j, may be used as a potential marker of HD, which might have important value for the diagnosis and prognosis of HD. It is reported that hsa-mir-10b-5p was activated in HD^11,21^ and contributed to the development of cancer metastasis^75–77^. Our results suggested that editing of hsa-mir-10b-5p might play a role in HD. More research is needed in the future to clarify the relationship between miRNA editing and/or mutation in HD and the pathogenesis of HD.

## Conclusions

An immense progress has been made in the regulation and role of miRNAs in a variety of nuerodegenarative disordres since the discovery of miRNAs in 1993^78,79^. However, miRNA editing events in Huntington’s disease have largely not been reported in previous studies. In our study, we analyzed 111 sRNA-seq sample data of human brain tissues, of which 28 were brain tissue samples with HD and 83 were healthy human brain tissue samples and found 1182 editing sites were identified by the MiRME algorithm, including 27 A-to-I, 4 C-to-U, 13 Other, 515 3’-A, 456 3’-U, 76 3’-Other, 55 5’-editing, 30 SNP, and 6 Pseudo sites. Furthermore, there were 129 editing events with significant differences in the brain tissues of HD patients and healthy individuals, the editing levels of 76 editing events were increased in HD, and 53 editing events were decreased. These changes may be related to the pathogenesis of HD.

One of the M/E sites, hsa-mir-10b_26_A_c, has higher editing level in HD and edited hsa-mir-10b-5p has higher expression level in HD. After comparing the targets of original and edited hsa-mir-10b-5p, we found that the 5’ edited hsa-mir-10b-5p may acquire as many as 308 new targets, including GTPBP10. GTPBP10 is often downregulated in HD (Fig. S1) and may deteriorate Huntington’s disease by affecting the synthesis of mitochondria and other related functions. Our results suggested that the increased editing level of hsa-mir-10b_26_A_c might aggravate Huntington’s disease by repressing GTPBP10.

## Methods

### Small RNA sequencing profiles used

We collected 111 sRNA-seq data in postmortem HD patients and healthy controls, including 28 prefrontal cortex of postmortem HD patients (HD-PC) and 83 brain samples of healthy ones, i.e., 38 prefrontal cortex (PC), 14 amygdala (Am), 6 frontal cortex (FC), 6 corpus callosum (CC), 3 astrocytes (As), 1 temporal neocortex gray matte (NG) and 15 unknown brain region (Unknown) samples. These 111 public data were downloaded from the NCBI SRA (National Center for Biotechnology Information, Sequence Read Archive database) database with their accession numbers in Table S1. The qualities of the obtained sRNA profiles were evaluated with the FastQC program^80^ (https://www.bioinformatics.babraham.ac.uk/projects/fastqc).

### Genomic sequences and miRNA annotations used

Human unmasked genomic sequences (hg38, GRCh38, Genome Reference Consortium Human Build 38) were downloaded from UCSC Gnome Browser^81^, of which the index of files was generated with the bowtie-build program in the Bowtie package^82^. The sequences and genomic positions of human miRNAs were downloaded from the miRBase (microRNA database, release 21)^83^.

### Identifying miRNA mutation and editing sites in HD

The 111 selected sRNA-seq profiles were analyzed using the MiRME pipeline as described in previously study with its default settings^36^. Briefly, the raw reads were done with the quality examination to keep the qualified reads of which the sequencing scores of 25 nucleotides have sequencing scores of 30 or higher. Then, the unique miRNA sequences of the remaining reads were obtained and counts of unique reads with more than 18 nucleotides were calculated. And the unique reads were aligned to pre-miRNAs using BLASTN (Nucleotide Basic Local Alignment Search Tool) with the options of “-S 1 -m 8 -e 0.01” and the reads mapped to pre-miRNAs were retrieved. Then, these reads mapped to pre-miRNAs were aligned to the genome using Bowtie (v1.0.0)^82^ with the options of “-a -best -S -v 1”. Then, the alignments of reads to genome were examined by the cross-mapping correction method^84^ to adjust the weights or percentages of a unique read at each of its genomic loci. In the main step, the MiRME algorithm with the default parameters was used to identify M/E sites in miRNAs from the sequences and structures of pre-miRNAs, the alignment of reads to the genome generated by Bowtie, the reads mapped to pre-miRNAs, the alignments of reads to pre-miRNAs generated by BLASTN, and the results of the cross-mapping correction method^84^. Then, the obtained results of different samples were combined by a separate program in the MiRME package (see details in^36,67^). The identified M/E sites were named by the miRNA name, the M/E position in pre-miRNA, original nucleotide in upper case, and edited/mutated nucleotide in lower case.

The criteria used to define significant M/E sites include: (i) the editing level is at least 5%; (ii) at least 10 reads support the editing event; (iii) the score threshold of sequencing reads is 30; (iv) a multiple-test corrected *P*-value (using the Benjamini and Hochberg method^85^) of smaller than 0.05. To remove M/E sites due to random sequencing errors, 1182 M/E sites that had significant editing level at least 7% of the 111 samples used in this study (8 samples) were kept in further analysis.

The identified M/E sites were compared to reported editing sites in miRNAs in the DARNED (DAtabase of RNa EDiting) database^86^, the RADAR (rigorously annotated database of A-to-I RNA editing database) database^87^ and literature^36,47,48,88,89^. Finally, the predicted M/E sites that belonged to A-to-I, C-to-U and other were manually examined.

The A-to-I and C-to-U editing sites were compared to those in *Macaca mulatta*^62^ and *Mus musculus*^31,47^, respectively. The editing sites of the same editing types that locate on the same positions of mature miRNAs of at least two different species were considered as conserved editing sites.

### Identifying SNPs in miRNAs

The identified M/E sites were compared to reported SNPs in miRNAs reported previouly^51^ (which was based on the dbSNP v137) and compared to the reported SNPs in the dbSNP (v151) database. Only sites that satisfied the following criteria were regarded as SNPs, (i) had the same genomic positions as the SNPs, (ii) had the same nucleotides as the alleles of the SNPs for both the original and changed nucleotides, and (iii) had editing levels of 100% in at least one of the 111 samples selected.

### Identifying miRNA M/E sites with different editing levels in HD

The editing levels of the 1182 miRNA M/E sites in the HD-PC (n = 28) and PC (n = 38) samples were compared with the Mann-Whitney *U*-tests. The obtained *P*-values were corrected with the Benjamini-Hochberg correction method^85^. M/E sites with multiple test corrected *P*-value smaller than 0.05 were regarded as having significantly different editing levels in HD-PC samples compared to PC samples.

### Predicting putative targets for the original and edited/mutated miRNAs

The targets of original and 5’ edited hsa-mir-10b-5p (hsa-mir-10b_26c) was predicted using the MiCPAR (MiRNA target prediction using Corrected PAR-CLIP) algorithm^67^ with its default parameters. Thetargets that had at least 1 PAR-CLIP (Photoactivatable Ribonucleoside-Enhanced Crosslinking and Immunoprecipitation) read with T-to-C (Thymidine to Cytidine) variation were kept in further analysis. As shown in Table S2, eleven PAR-CLIP sequencing profiles were downloaded from the NCBI SRA database using the series accession number SRP002487(seven from HEK293 cells stably expressing FLAG/HA-tagged AGO (Argonaute) proteins include AGO1, AGO2, AGO3, and AGO4^90^) and SRP018015 (four from HEK293 cell lines stably expressing HIS/FLAG/HA-tagged AGO1 or AGO2^91^). Then, the original reads in the 11 PAR-CLIP sequencing files are screened and processed to obtain qualified reads. The remaining reads in these 11 profiles were combined and used in the identification of miRNA targets with the MiCPAR algorithm^67^. The annotation of NCBI RefSeq genes in the GTF (Gene transfer format) file, the mRNA sequences of NCBI RefSeq genes (version hg38) and soft-masked genome sequences of human (version GRCh38) were downloaded from the UCSC Genome Browser (University of California, Santa Cruz Genome Browser)^92^ and used as inputs of the MiCPAR algorithm. The targets with at least 1 PAR-CLIP read with T-to-C variation were kept for further analysis^64^.

We selected two SNPs, hsa-mir-499a_73_A_g and hsa-mir-3622a_21_G_a, which located in the seed regions of mature miRNAs, i.e., the first to eighth nucleotide, from 30 SNPs in miRNAs (Table S6). We then used the MiCPAR algorithm^67^ to predict the targets of the two miRNAs for both original and mutated forms. The targets that had at least 1 PAR-CLIP read with T-to-C variation were kept in further analysis.

### GO term and KEGG pathway analysis for hsa-mir-10b_26_A_c

After comparing the targets of the original and 5’ edited hsa-mir-10b-5p (hsa-mir-10b_26c), new targets of hsa-mir-10b_26c were identified. KOBAS (version 3.0)^68^ was used to perform GO items and KEGG pathways^93–95^ analysis on the new targets of hsa-mir-10b_26c. The significantly enriched GO items and KEGG pathways were filtered with the criteria of multiple test corrected *P*-values smaller than 0.05.

## Supplementary Information

Supplementary Information 1.

Supplementary Information 2.

## Data Availability

The 111 published sRNA-seq profiles were retrieved from the NCBI SRA database under the accession numbers listed in Table S1.
