# Supplementary material for: MicroRNA editing patterns in Huntington’s disease
Source: Sci Rep. 2022 Feb 24;12:3173. doi: 10.1038/s41598-022-06970-6 (PMC8873361; doi:10.1038/s41598-022-06970-6)
Supplement: Supplementary file 1 — Supplementary Information 1. [file 41598_2022_6970_MOESM1_ESM.pdf]

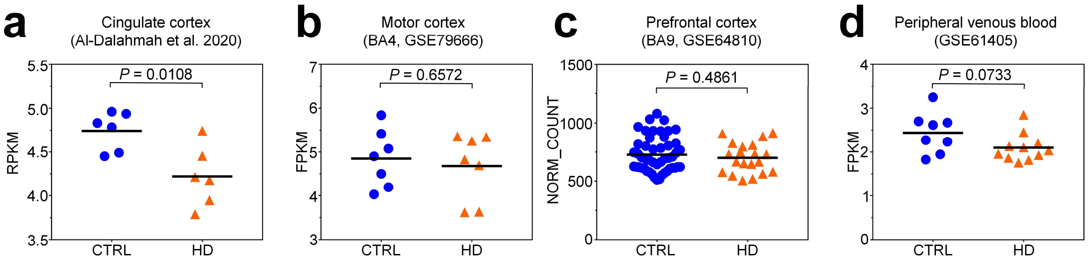

Figure S1. Comparisons of expression level of GTPBP10 in different brain regions of HD patients (HD) and normal controls (CTRL). *P*-values were calculated with two tailed *t*-tests. (a) GTPBP10 is significantly down-regulated in the cingulate cortex regions of HD patients compared to normal controls. The data was from AI-Dalahmah et al. 2020[1]. (b) GTPBP10 is down-regulated in motor cortex (BA4) regions of HD patients compared to normal controls (data from GSE79666)[2]. (c) GTPBP10 is down-regulated in HD-PC (BA9) compared to normal controls (data from GSE64810)[3]. (d) GTPBP10 is down-regulated in peripheral venous blood samples of HD patients compared to normal controls (data from GSE61405)[4].

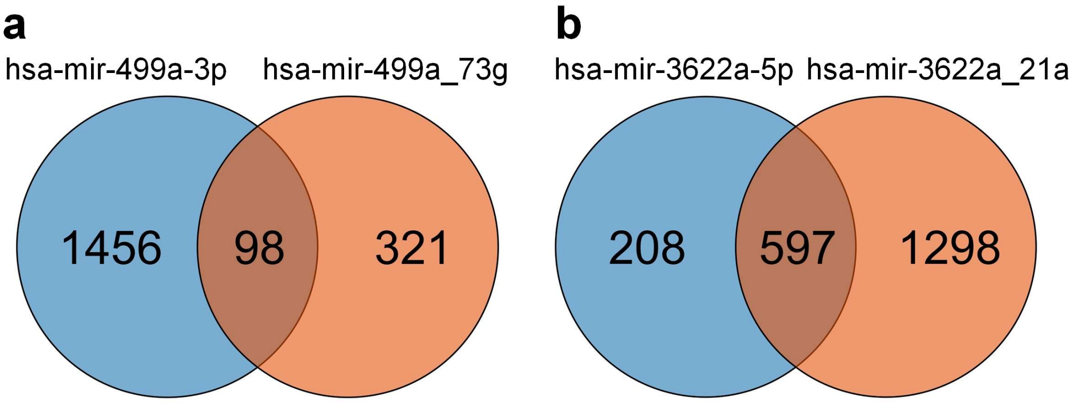

Figure S2. The comparisons for the targets of the original and mutated hsa-mir-499a-3p (hsa-mir-499a_73g) and hsa-mir-3622a-5p (hsa-mir-3622a_21a), respectively. (a) The targets of hsa-mir-499a-3p and hsa-mir-499a_73g. (b). The targets of hsa-mir-3622a-5p and hsa-mir-3622a_21a.

References

1. Al-Dalahmah, O., et al., *Single-nucleus RNA-seq identifies Huntington disease astrocyte states.* Acta Neuropathol Commun, 2020. **8**(1): p. 19.
2. Lin, L., et al., *Transcriptome sequencing reveals aberrant alternative splicing in Huntington's disease.* Hum Mol Genet, 2016. **25**(16): p. 3454-3466.
3. Labadorf, A., et al., *RNA Sequence Analysis of Human Huntington Disease Brain Reveals an Extensive Increase in Inflammatory and Developmental Gene Expression.* PLoS One, 2015. **10**(12): p. e0143563.
4. Andrade-Navarro, M.A., et al., *RNA Sequencing of Human Peripheral Blood Cells Indicates Upregulation of Immune-Related Genes in Huntington's Disease.* Front Neurol, 2020. **11**: p. 573560.
